# Roles of the Src Tyrosine Kinases Lck and Fyn in Regulating γδTCR Signal Strength

**DOI:** 10.1371/journal.pone.0008899

**Published:** 2010-01-26

**Authors:** Renee M. Laird, Sandra M. Hayes

**Affiliations:** Department of Microbiology and Immunology, SUNY Upstate Medical University, Syracuse, New York, United States of America; Oklahoma Medical Research Foundation, United States of America

## Abstract

Lck and Fyn, members of the Src family of tyrosine kinases, are key components of the αβTCR-coupled signaling pathway. While it is generally accepted that both Lck and Fyn positively regulate signal transduction by the αβTCR, recent studies have shown that Lck and Fyn have distinct functions in this signaling pathway, with Lck being a positive regulator and Fyn being a negative regulator of αβTCR signal transduction. To determine whether Lck and Fyn also differentially regulate γδTCR signal transduction, we analyzed γδ T cell development and function in mice with reduced Lck or Fyn expression levels. We found that reducing Lck or Fyn levels altered the strength of the γδTCR signaling response, with low levels of Lck weakening γδTCR signal strength and low levels of Fyn augmenting γδTCR signal strength. These alterations in γδTCR signal strength had profound effects not only on αβ/γδ lineage choice, but also on γδ thymocyte maturation and γδ T cell effector function. These results indicate that the cellular levels of Lck and Fyn play a role in regulating the strength of the γδTCR signaling response at different stages in the life of the γδ T cell.

## Introduction

Signaling by the TCR is required at multiple stages in the life of a T cell. In the thymus, TCR signaling is necessary for lineage commitment and repertoire selection, while in the periphery, TCR signaling is necessary for maintenance of the peripheral T cell pool and for activation and differentiation of mature T cells. Lck and Fyn, two members of the Src family of tyrosine kinases (SFKs), are involved in initiating the TCR-coupled signaling cascade [1], [2]. Following TCR engagement, Lck and/or Fyn phosphorylate the tyrosines within the ITAMs of the CD3 and TCRζ chains. This proximal signaling event leads to the recruitment of other signaling molecules to the TCR signaling complex and to the subsequent activation of signaling pathways that ultimately lead to the nucleus and initiation of gene transcription.

It is generally accepted that both Lck and Fyn positively regulate signal transduction by the αβTCR because, in the absence of either one of these SFKs, αβTCR signaling responses are impaired following anti-CD3 mAb stimulation [3]–[9]. However, it has also been shown that Lck and Fyn localize to different subcellular compartments [10], [11] and have different substrates [11], [12], suggesting that they have discrete functions during αβ T cell activation. This idea is supported by the disparate phenotypes of Lck- and Fyn-deficient mice. In Lck^−/−^ mice, thymus cellularity is severely reduced, thymocyte development is almost completely blocked at the CD4^+^CD8^+^ (double-positive; DP) stage, and very few mature αβTCR^+^ cells are detected in peripheral lymphoid tissues [12]–[14]. In contrast, Fyn^−/−^ mice exhibit a mild defect in αβ T cell development, as shown by the fact that Fyn^−/−^ thymocytes, when *in vitro* stimulated, do not flux calcium or proliferate as well as wild-type (WT) thymocytes [3], [5]. Despite this signaling defect in the thymus, equivalent numbers of αβ T cells are found in the periphery of Fyn^−/−^ and WT mice [3], [5].

While recent studies have confirmed that Lck functions primarily as a positive regulator of αβTCR signaling [15]–[17], evidence is accumulating in support of Fyn acting as a negative regulator of αβTCR signaling. First, it has been shown that Fyn is responsible for phosphorylating the adaptor protein, phosphoprotein associated with glycolipid-enriched membranes or PAG, in both resting thymocytes and T cells [11]. Once phosphorylated, PAG then recruits Csk, an inhibitor of SFKs [18]. Recruitment of Csk to phosphorylated PAG is required for optimal Csk kinase activity because, in the absence of Fyn, there is reduced phosphorylation of PAG and reduced Csk kinase activity [11]. Therefore, by indirectly controlling the activity of the inhibitor Csk, Fyn may negatively regulate the activation threshold of αβ T cells [11]. It has also been shown that CD8^+^ T cells from Fyn^−/−^ F5 αβTCR Tg mice are hyperresponsive in comparison to CD8^+^ T cells from WT F5 αβTCR Tg mice following *in vitro* stimulation with peptide and APCs [19]. This hyperresponsiveness is manifested as enhanced proliferation, increased IL-2 production and more effective cytolytic activity [19]. CD4^+^ T cells from Fyn^−/−^ DO11.10 αβTCR Tg mice, however, do not display increased proliferation compared to CD4^+^ T cells from WT DO11.10 αβTCR Tg mice when stimulated, either *in vitro* or *in vivo*, with peptide and APCs [20]. Nonetheless, when activated under the appropriate priming conditions, CD4^+^ T cells from Fyn^−/−^ DO11.10 αβTCR Tg mice produce significantly more IL-4 or IFNγ than CD4^+^ T cells from WT DO11.10 αβTCR Tg mice [20]. Taken together, these findings suggest that Fyn negatively regulates the αβTCR signaling response.

Since studies investigating the functions of Lck and Fyn have focused primarily on αβ T cells, it is not known whether their functional dichotomy is observed in only αβ T lineage cells or in both αβ and γδ T lineage cells. Analyses of Lck^−/−^ and Lck^−/−^ Fyn^−/−^ mice have in fact revealed differences in the requirements for these SFKs in αβ and γδ T cell development. In Lck^−/−^ mice, the number of thymic and peripheral γδ T cells is only modestly reduced compared to their numbers in WT mice [13], [14], [21]. Moreover, in Lck^−/−^ Fyn^−/−^ mice, in which αβ T cell development is completely abrogated, a small number of γδTCR^+^ cells do develop and can be detected in secondary lymphoid tissues, the small intestine, and the epidermis [21], [22]. These differential requirements for Lck and Fyn in αβ and γδ T cell development suggest that these SFKs may have different functions in αβ- and γδTCR signal transduction. To investigate this, we evaluated the individual roles of Lck and Fyn in the development and function of γδ lineage cells. Here, we report that Lck and Fyn expression levels vary in γδ lineage cells depending on their stage in development, with thymic γδ T cells expressing relatively high levels of Lck and Fyn and peripheral γδ T cells expressing relatively low levels of Lck and Fyn. These differences in the cellular levels of Lck and Fyn play a role in regulating the strength of the γδTCR signaling response at the different developmental stages because, when we reduced Lck or Fyn expression levels by using Lck^+/−^ and Fyn^+/−^ mice, we observed significant effects on αβ/γδ lineage choice, γδ thymocyte maturation, and γδ T cell effector function. Moreover, because reducing the levels of Lck or Fyn altered the γδTCR signaling response, such that low Lck levels weakened γδTCR signal strength and low Fyn levels augmented γδTCR signal strength, we conclude that Lck and Fyn have similar functions in αβ- and γδTCR signal transduction, with Lck serving to amplify the TCR signal and Fyn serving to dampen the TCR signal.

## Results

### Expression Pattern of Lck and Fyn in γδ Lineage Cells

**Figure 1:**
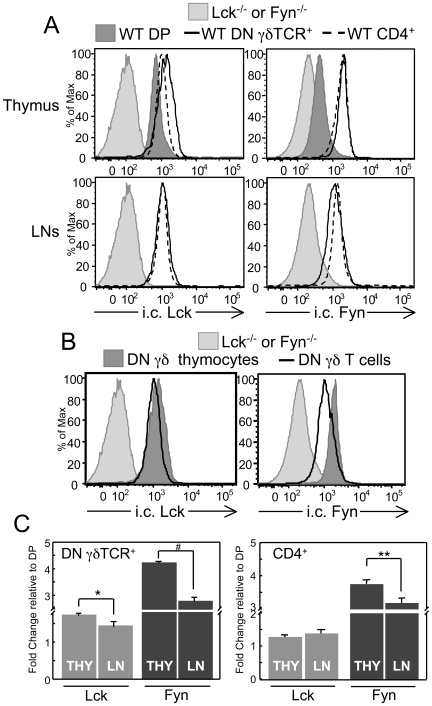
Flow cytometric analysis of the intracellular levels of Lck and Fyn in γδ lineage cells. A. Histograms show representative staining of the i.c. levels of Lck and Fyn in gated populations of DP thymocytes and of thymic and LN CD4^+^ CD3^+^ and DN γδTCR^+^ cells from WT (B6) mice. Staining of cells from Lck^−/−^ and Fyn^−/−^ mice are shown as negative controls for i.c. staining of Lck and Fyn, respectively. B. Comparison of the relative expression levels of Lck and Fyn in gated DN γδTCR^+^ thymocytes and LN cells. C. Quantifying the change in the relative expression levels of Lck and Fyn in DN γδTCR^+^ thymocytes and LN cells and, for comparison, CD4^+^ CD3^+^ thymocytes and LN cells. Lck and Fyn expression levels in immature and mature subsets were normalized to those of DP thymocytes, as this population had, in every experiment, consistently lower levels of Lck and Fyn than any other thymocyte or T cell subset (see A). Data are presented as fold change relative to DP thymocytes (set to 1). Data are representative of at least 6 independent experiments. Bars represent mean ± SEM. *p≤0.05, **p≤0.01, #*p≤0.001*.

Although it is generally accepted that γδ T cells express Lck and Fyn, this idea is based more on indirect evidence from studies investigating γδ T cell development in Lck- or Fyn-deficient mice [13], [14], [21], [23], [24] than on a direct demonstration of expression [25]. To resolve this, we developed an intracellular (i.c.) flow cytometric assay to measure and compare the relative levels of Lck and Fyn in αβ and γδ lineage cells from wild-type (WT) mice. Using Lck^−/−^ and Fyn^−/−^ cells as negative staining controls, we found that both Lck and Fyn are expressed in DN γδ thymocytes and peripheral DN γδ T cells (Fig. 1A). On average, DN γδ thymocytes expressed Lck and Fyn at higher levels than DP and mature CD4^+^ thymocytes, whereas peripheral DN γδ T cells expressed Lck at levels comparable to those in CD4^+^ T cells and Fyn at levels lower than those in CD4^+^ T cells (Fig. 1A). When Lck and Fyn expression levels were compared between thymic and peripheral γδ T cells, we found that both SFKs are expressed at significantly higher levels in DN γδ thymocytes than in DN γδ T cells (Fig. 1B–C). This finding suggested that the expression levels of both Lck and Fyn are down-regulated once γδ T cells emigrate from the thymus to the secondary lymphoid organs. This same phenomenon was also observed for αβ lineage cells; however, the degree of reduction in Lck and Fyn expression levels between the thymus and LN was greater for γδ lineage cells than αβ lineage cells (Fig. 1C and data not shown). Taken together, these data indicated that immature and mature γδ lineage cells express Lck and Fyn and that the expression of these SFKs is dynamic during γδ T cell development and maturation.

### Polyclonal γδ T Cell Development in Lck^+/−^ and Fyn^+/−^ Mice

**Figure 2:**
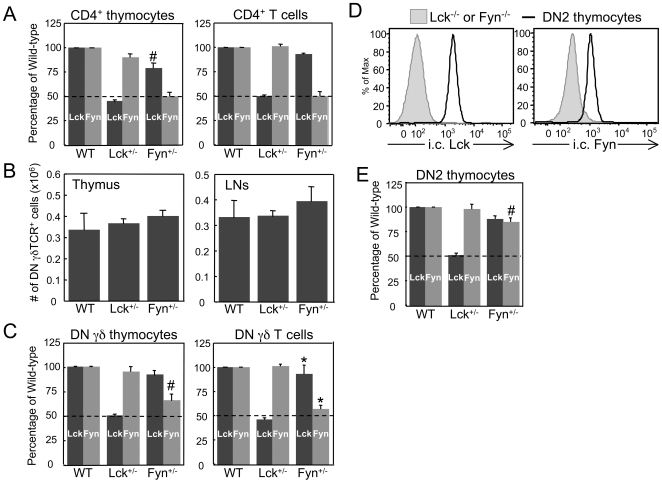
Effect of reducing Lck or Fyn levels on polyclonal γδ T cell development. A. Demonstration of the reduction of Lck or Fyn levels in CD4^+^ thymocytes and LN cells from Lck^+/−^ and Fyn^+/−^ mice. The MFI of the i.c. levels of Lck and Fyn in CD4^+^ lineage cells from heterozygous mice are expressed as a percentage of the MFI of the i.c. levels of Lck and Fyn in CD4^+^ lineage cells from WT mice. A dashed line marks the expected 50% reduction in WT Lck and Fyn levels. B. Number of DN γδ thymocytes and LN γδ T cells in WT, Lck^+/−^, and Fyn^+/−^ mice. Data represent at least 6 mice per genotype. C. Quantifying the reduction of Lck and Fyn expression levels in DN γδTCR^+^ thymocytes and LN cells from Lck^+/−^ and Fyn^+/−^ mice. The MFI of the i.c. levels of Lck and Fyn in γδ lineage cells from heterozygous mice are expressed as a percentage of the MFI of the i.c. levels of Lck and Fyn in γδ lineage cells from WT mice. A dashed line marks the expected 50% reduction in WT Lck and Fyn levels. D. Relative expression levels of Lck and Fyn in WT DN2 (lin^−^ CD25^+^ CD44^+^) thymocytes, where lin^−^ is defined as CD4^−^ CD8^−^ CD11b^−^ TCRβ^−^ TCRγδ^−^ CD19^−^ NK1.1^−^ IA^b−^ Ly6-G/Ly6-C^−^. E. Quantifying the reduction of Lck and Fyn expression levels in DN2 thymocytes from Lck^+/−^ and Fyn^+/−^ mice. The MFI of the i.c. levels of Lck and Fyn in DN2 thymocytes from heterozygous mice are expressed as a percentage of the MFI of the i.c. levels of Lck and Fyn in DN2 thymocytes from WT mice. A dashed line marks the expected 50% reduction in WT Lck and Fyn levels. In A, B, C, and E, the bars represent mean ± SEM. *p≤0.05, **p≤0.01, #*p≤0.001*.

Because DN γδ thymocytes expressed higher levels of Lck and Fyn than mature DN γδ T cells, we sought to determine whether high levels of Lck or Fyn were required for γδ lineage commitment and/or development in the thymus. To investigate this, we reduced the expression levels of Lck or Fyn during T cell development by using Lck^+/−^ and Fyn^+/−^ mice. To verify that protein expression was reduced in the heterozygous mice, we compared the relative expression levels of Lck and Fyn in immature and mature CD4^+^ lineage cells from Lck^+/−^ and Fyn^+/−^ mice with those from WT mice. As expected, we observed a 50% reduction in Lck expression levels in Lck^+/−^ mice and a 50% reduction in Fyn expression levels in Fyn^+/−^ mice (Fig. 2A). In addition, there was no compensatory increase in the expression of one SFK when expression of the other SFK was reduced (Fig. 2A).

When γδ T cell development was analyzed in Lck^+/−^ and Fyn^+/−^ mice, we observed no significant difference in the number of DN γδ TCR^+^ cells in the thymus and lymph nodes (LNs) of these mice compared to WT mice (Fig. 2B). Moreover, phenotypic analysis of the DN γδ thymocytes and mature DN γδ T cells from WT, Lck^+/−^ and Fyn^+/−^ mice revealed no appreciable differences in Vγ usage, TCRγδ surface levels and cell surface phenotype (data not shown), indicating that reducing the expression levels of Lck or Fyn resulted in no apparent defect in the development of polyclonal γδ T cells.

Because γδ T cell development appeared not to be affected in Lck^+/−^ and Fyn^+/−^ mice, we examined the levels of Lck and Fyn in γδ lineage cells of the heterozygous mice to determine whether they were reduced by 50% as they were in αβ lineage cells (Fig. 2A and C). We found that Lck levels were indeed reduced by 50% in thymic and peripheral γδ lineage cells from Lck^+/−^ mice (Fig. 2C). Fyn levels, on the other hand, were reduced to ∼60% in immature and mature γδ lineage cells from Fyn^+/−^ mice, which is significantly different from the expected 50% (Fig. 2C). Since Fyn expression levels in γδ lineage cells were not reduced to the expected 50% in Fyn^+/−^ mice, we determined whether the thymic precursors in Lck^+/−^ and Fyn^+/−^ mice displayed a 50% reduction in Lck and Fyn expression levels, respectively. To accomplish this, we compared Lck and Fyn expression levels in lineage-negative CD44^+^CD25^+^ (DN2) thymocytes from WT, Lck^+/−^ and Fyn^+/−^ mice, as this thymocyte subset contains precursors that have the potential to develop into αβ or γδ lineage cells [26], [27]. We found that, while Lck expression levels were reduced to 50% in Lck^+/−^ DN2 thymocytes, Fyn expression levels were only reduced to 85% in Fyn^+/−^ DN2 thymocytes (Fig. 2D and E). These data demonstrated that although Fyn levels are reduced in thymic precursors and γδ lineage cells from Fyn^+/−^ mice, they are not reduced by 50% as they are in αβ lineage cells.

### Effect of Reducing Lck or Fyn Levels on the Commitment and Development of γδ Lineage Cells

Another reason why we may not have observed any defects in γδ T cell development in Lck^+/−^ and Fyn^+/−^ mice is because thymocyte development and selection are able to compensate for alterations in γδTCR signal transduction, which may result from reductions in Lck or Fyn expression. To address this, we mated a γδTCR transgene onto Lck^+/−^ and Fyn^+/−^ genetic backgrounds to determine whether fixing the specificity of the γδTCR revealed defects in αβ/γδ lineage choice and/or γδ T cell development. For these experiments, we used the Vγ6/Jγ1/Cγ1 and Vδ1/Dδ1/Jδ2/Cδ transgenic (γδTCR Tg) mouse [28], which we have previously used to study αβ/γδ lineage choice, γδ T cell development and γδTCR signal transduction [29]–[31]. It is important to note that, although Vγ6/Vδ1^+^ γδ T cells are only generated in the fetal thymus of a WT (non-γδTCR Tg) mouse [28], the Vγ6/Vδ1^+^ γδ T cells generated in the γδTCR Tg mouse represent adult γδ T cells, as they express the panel of γδ-biased genes [31] typical of adult but not fetal γδ T cell populations [32]. Moreover, one of the advantages of using this γδTCR Tg mouse model to study αβ/γδ lineage choice is that the rearranged TCRγ and –δ chains are expressed early during T cell development prior to TCRβ expression and, as a consequence, the αβ/γδ lineage decision is mediated exclusively by the γδTCR [29].

**Figure 3:**
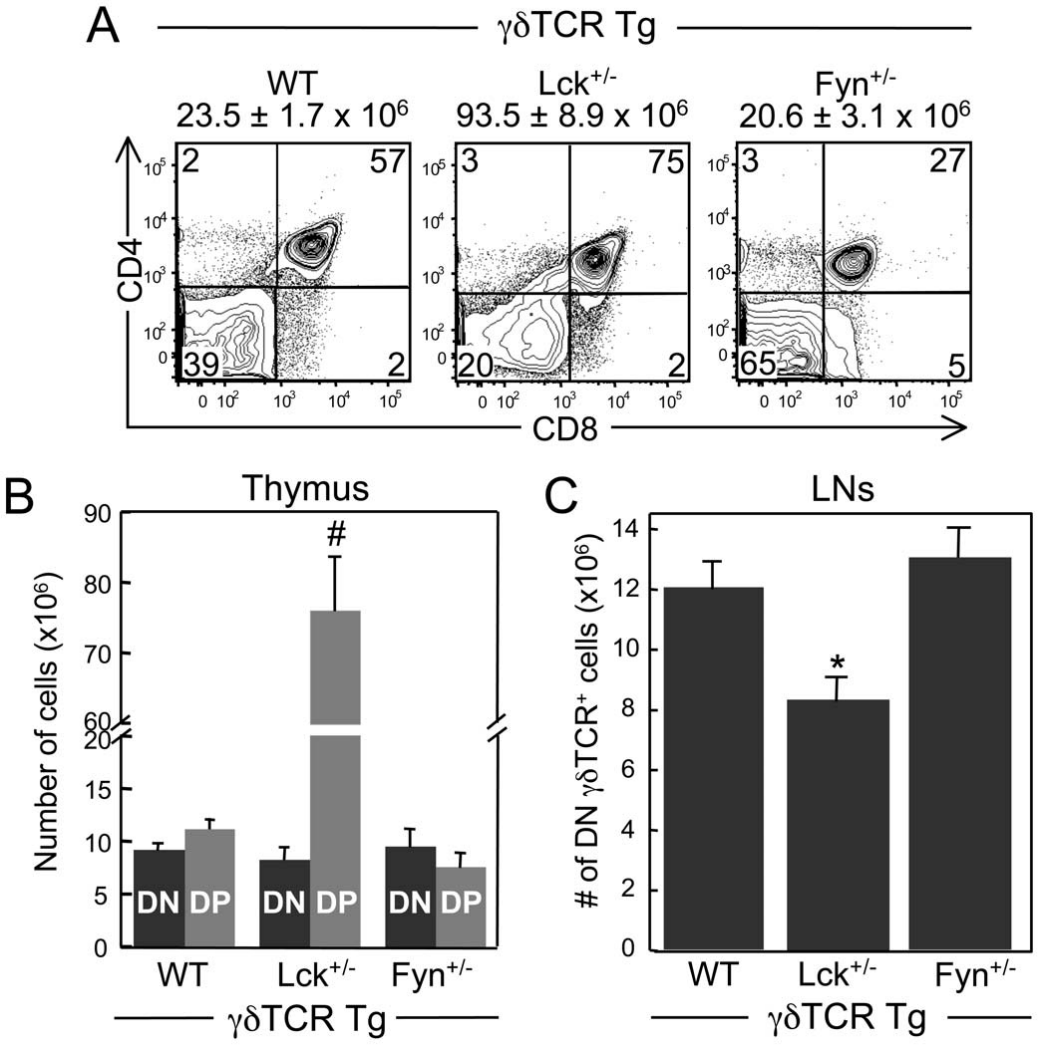
Effect of reducing Lck or Fyn levels on αβ/γδ lineage commitment and γδ T cell development. A. Dot plots show representative CD4 versus CD8 staining profiles for WT γδTCR Tg, Lck^+/−^ γδTCR Tg, and Fyn^+/−^ γδTCR Tg thymocytes. Numbers in the quadrants represent percentage of cells in each quadrant. The mean thymus cell number ± SEM for each genotype are displayed above the respective two-color plot. B. Mean number of DN (DN γδTCR^+^; γδ lineage) and DP (αβ lineage) thymocytes in WT γδTCR Tg, Lck^+/−^ γδTCR Tg and Fyn^+/−^ γδTCR Tg mice. Data represent at least 6 mice per genotype. C. Mean number of DN γδ T cells in the LNs of WT γδTCR Tg, Lck^+/−^ γδTCR Tg and Fyn^+/−^ γδTCR Tg mice. Data represent at least 5 mice per genotype. In B and C, the bars represent mean ± SEM. *p≤0.05, #*p≤0.001*.

By fixing the specificity of the γδTCR, we observed significant effects on the αβ/γδ lineage fate decision when the levels of Lck but not Fyn were reduced. In WT γδTCR Tg mice, equivalent numbers of DN γδTCR^+^ thymocytes (γδ lineage) and DP thymocytes (αβ lineage cells) are generated (Fig. 3A–B). Reducing Lck expression resulted in a striking 4-fold increase in thymus cell number compared to WT γδTCR Tg mice, most likely due to the significant increase in the percentage of DP thymocytes (Fig. 3A). Consequently, the number of αβ lineage cells in Lck^+/−^ γδTCR Tg mice was significantly higher than that in WT γδTCR Tg mice (Fig. 3B). These findings are consistent with those of a previous study, in which γδTCR-dependent generation of DP thymocytes but not of DN γδTCR^+^ thymocytes was observed in the absence of Lck expression [33]. In contrast, reducing Fyn levels had no effect on total thymus cell number. Nonetheless, we did observe a decrease in both the percentage and number of DP thymocytes in Fyn^+/−^ γδTCR Tg mice, although these differences were not statistically significant from the percentage and number of DP thymocytes in WT γδTCR Tg mice (Fig. 3A–B). Interestingly, despite the changes in the numbers of DP thymocytes in the heterozygous mice, the numbers of DN γδTCR^+^ thymocytes, or γδ lineage cells, in Lck^+/−^ γδTCR Tg and Fyn^+/−^ γδTCR Tg mice were comparable to their number in WT γδTCR Tg mice (Fig. 3A–B).

It has previously been shown that genetic manipulation of γδTCR signal strength affects αβ/γδ lineage choice in a consistent manner. Namely, when the γδTCR signaling response is strengthened, γδ lineage fate is favored and, conversely, when the γδTCR signaling response is weakened, αβ lineage fate is favored [29], [33]. Our finding that the number of αβ lineage cells was significantly increased in Lck^+/−^ γδTCR Tg mice compared to WT γδTCR Tg mice suggested that reducing Lck levels weakened γδTCR signal strength. Surprisingly, this increase in the number of DP thymocytes was not accompanied by a corresponding decrease in the number of DN γδ thymocytes in Lck^+/−^ γδTCR Tg (Fig. 3B). We reasoned that the number of DN γδ thymocytes in Lck^+/−^ γδTCR Tg mice may not reflect the number of thymocytes that adopted the γδ fate but instead reflected an expansion of the thymocytes that already adopted the γδ fate. To investigate this, we compared the proliferative status of DN γδ thymocytes in WT γδTCR Tg, Lck^+/−^ γδTCR Tg, and Fyn^+/−^ γδTCR Tg mice by measuring their expression of the Ki-67 Ag, which is a marker of actively cycling cells [34], [35]. We found that the frequency of Ki67^+^ DN γδ thymocytes in Lck^+/−^ γδTCR Tg mice was significantly higher than the frequency of Ki67^+^ DN γδ thymocytes in WT γδTCR Tg and Fyn^+/−^ γδTCR Tg mice (Table 1). These data suggested that the number of DN γδ thymocytes in Lck^+/−^ γδTCR Tg mice does not reflect the number of thymocytes that adopted the γδ fate.

**Table 1:** Percentage of Ki-67^+^ DN γδTCR^+^ thymocytes^a^.

Genotype	% Ki-67^+^ DN γδTCR^+^
WT γδTCR Tg	32.6±1.2
Lck^+/−^ γδTCR Tg	43.7±6.0**
Fyn^+/−^ γδTCR Tg	32.3±1.8

*^a^*Ki-67 expression marks cells in late G_1_ phase through mitosis and is used as marker of active cell cycling.

**p≤0.01.

**Figure 4:**
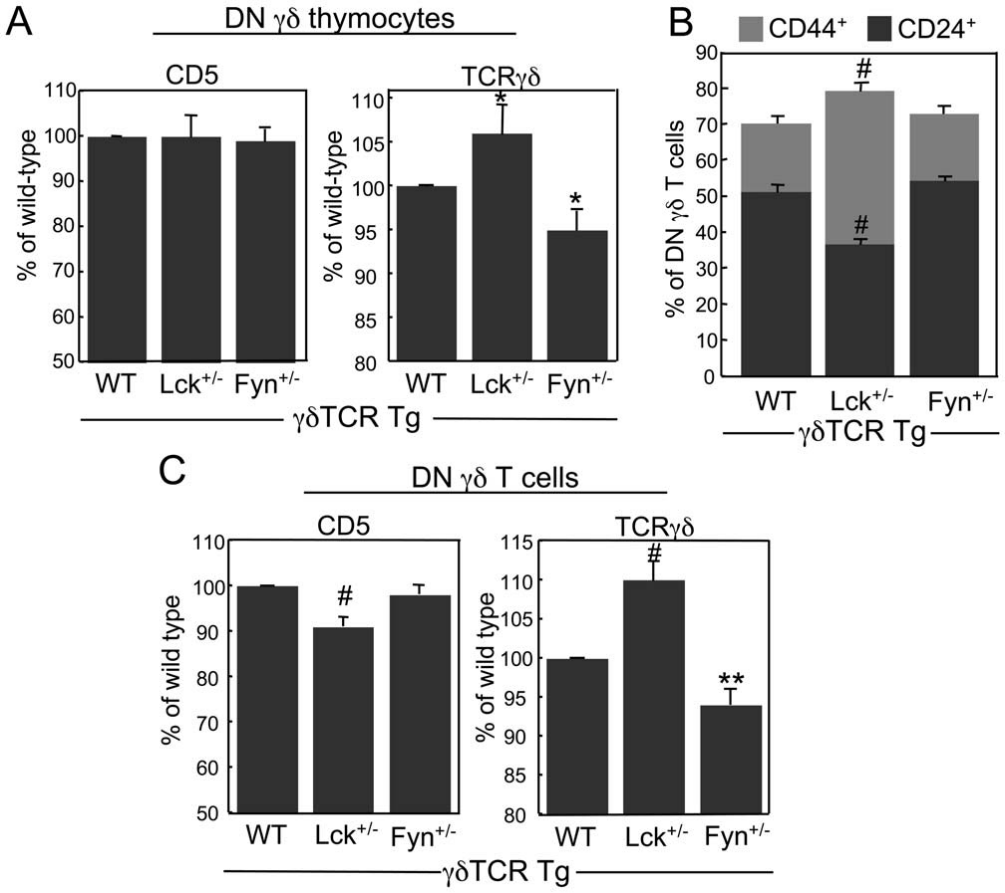
Phenotypic analysis of γδ lineage cells from Lck^+/−^ γδTCR Tg and Fyn^+/−^ γδTCR Tg mice. A. Comparison of CD5 and TCRγδ surface levels on DN γδ thymocytes from WT γδTCR Tg, Lck^+/−^ γδTCR Tg and Fyn^+/−^ γδTCR Tg mice. MFIs of CD5 and TCRγδ surface levels on DN γδ thymocytes from heterozygous mice are presented as a percentage of the MFIs of CD5 and TCRγδ surface levels on DN γδ thymocytes from WT γδTCR Tg mice. Data represent at least 6 mice per genotype. B. Percentage of CD24^+^ and CD44^+^ γδ T cells in WT γδTCR Tg, Lck^+/−^ γδTCR Tg and Fyn^+/−^ γδTCR Tg mice. Data represent at least 3 mice per genotype. C. Comparison of CD5 and TCRγδ surface levels on DN γδ T cells from the LNs of WT γδTCR Tg, Lck^+/−^ γδTCR Tg and Fyn^+/−^ γδTCR Tg mice. MFIs of CD5 and TCRγδ surface levels on peripheral DN γδ T cells from heterozygous mice are presented as a percentage of the MFIs of CD5 and TCRγδ surface levels on peripheral DN γδ T cells from WT γδTCR Tg mice. Data represent at least 5 mice per genotype. In A, B, and C, the bars represent mean ± SEM. *p≤0.05, **p≤0.01, #*p≤0.001*.

Next, we compared the expression levels of the γδTCR and CD5 on the surface of DN γδ thymocytes from WT γδTCR Tg, Lck^+/−^ γδTCR Tg, and Fyn^+/−^ γδTCR Tg mice to gauge the effects of reducing Lck or Fyn levels on the phenotype of the cells choosing the γδ lineage. When we examined γδTCR and CD5 surface levels on the DN γδ thymocytes that were generated in WT γδTCR Tg, Lck^+/−^ γδTCR Tg and Fyn^+/−^ γδTCR Tg mice, we found that DN γδ thymocytes in the three genotypes expressed different levels of the γδTCR but equivalent levels of CD5. Specifically, Lck^+/−^ γδ thymocytes expressed significantly higher levels of the γδTCR than WT γδ thymocytes, whereas Fyn^+/−^ γδ thymocytes expressed significantly lower levels of the γδTCR than WT γδ thymocytes (Fig. 4A). These data suggested that reducing Lck expression levels weakened γδTCR signal strength to the extent that immature thymocytes expressing relatively high levels of the γδTCR adopted the γδ fate. Conversely, reducing Fyn expression levels augmented γδTCR signal strength to where immature thymocytes expressing relatively low levels of the γδTCR adopted the γδ fate.

To investigate the effects of reducing the levels of Lck or Fyn on the maturation of γδ T cells in the thymus and their subsequent ability to migrate to the periphery, we enumerated DN γδ T cells in the LNs of WT γδTCR Tg, Lck^+/−^ γδTCR Tg, and Fyn^+/−^ γδTCR Tg mice. Compared to WT γδTCR Tg mice, we observed a significant decrease in the number of DN γδ T cells in Lck^+/−^ γδTCR Tg mice but not in the number of DN γδ T cells in Fyn^+/−^ γδTCR Tg mice (Fig. 3C). Consistent with the lower numbers of peripheral γδ T cells in Lck^+/−^ γδTCR Tg mice was the finding that there were fewer DN γδ T cells in these mice that expressed CD24, a marker of recent thymic emigrant γδ T cells [36], and that there were more cells that expressed CD44, a marker of activated cells, memory cells, and/or cells undergoing homeostatic proliferation [36]–[38] (Fig. 4B). Moreover, we found that the γδTCR and CD5 surface levels that were noted among the DN γδTCR^+^ thymocytes from the three genotypes were maintained on their respective peripheral γδ T cells, with the exception that Lck^+/−^ γδ T cells expressed significantly lower levels of CD5 than WT γδ T cells (Fig. 4C). Therefore, although DN γδ thymocytes were generated in Lck^+/−^ γδTCR Tg mice in numbers comparable to WT γδTCR Tg mice, these mice had reduced numbers of mature DN γδ T cells. These data indicated that a reduction in Lck levels but not Fyn levels affects the maturation and/or survival of thymic γδ T cells.

### Effect of Reducing Lck or Fyn Levels on γδ T Cell Effector Fate and Function

**Figure 5:**
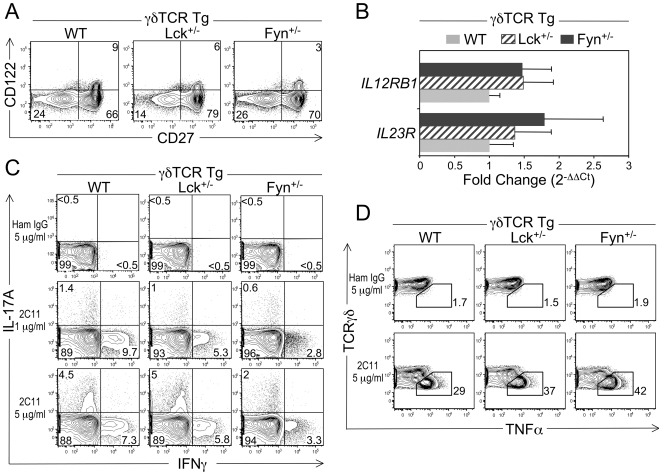
Effect of reducing Lck or Fyn levels on γδ T cell effector fate and function. A. Dot plots showing representative CD122 versus CD27 staining profiles for DN γδTCR^+^ LN cells from WT γδTCR Tg, Lck^+/−^ γδTCR Tg and Fyn^+/−^ γδTCR Tg mice. Numbers in the quadrants represent percentage of cells in that quadrant. The percentage of CD122^+^ DN γδ T cells is significantly lower in Fyn^+/−^ γδTCR Tg mice than in WT γδTCR Tg mice, *p≤0.05*. Data are representative of at least 6 mice per genotype. B. Quantitative real-time RT-PCR analysis of the relative transcript levels of *IL12RB1* and *IL23R* in purified peripheral DN γδ T cells from WT γδTCR Tg, Lck^+/−^ γδTCR Tg and Fyn^+/−^ γδTCR Tg mice. Data are normalized to *GAPDH* and are presented as fold change over WT γδ T cells (set to 1). Bars represent mean ± SEM. Data represent 3 mice per genotype. C. Comparison of IL-17 and IFNγ production by DN γδTCR^+^ LN cells from WT γδTCR Tg, Lck^+/−^ γδTCR Tg and Fyn^+/−^ γδTCR Tg mice. LN cells from the three genotypes were *in vitro* stimulated with 1 or 5 µg/ml of immobilized anti-CD3 mAb or 5 µg/ml of immobilized hamster IgG. 16 h later, cells were harvested and cytokine production was assayed by i.c. flow cytometric analysis. Dot plots show representative i.c. staining for IFNγ versus IL-17 in gated DN γδTCR^+^ cells. Numbers in the quadrants represent percentage of cells in that quadrant. Data shown are representative of at least 3 mice per genotype. D. Comparison of the ability of DN γδ T cells from WT γδTCR Tg, Lck^+/−^ γδTCR Tg and Fyn^+/−^ γδTCR Tg to produce TNFα. Lymph node cells from each genotype were *in vitro* stimulated with 5 µg/ml of immobilized anti-CD3 mAb or 5 µg/ml of immobilized hamster IgG. 48 h later, cytokine production was measured by i.c. flow cytometry. Dot plots show representative staining for i.c. TNFα versus TCRγδ in gated DN γδTCR^+^ cells. The percentage of TNFα-producing γδ T cells for each genotype is shown. Data are representative of at least 4 mice per genotype.

γδ T cell effector fate has been shown to segregate with expression of specific surface antigens, specifically CD122^+^ and/or CD27^+^ γδ T cells preferentially produce IFNγ [39], [40], whereas IL-23R^+^ γδ T cells preferentially produce IL-17 [41]–[43]. Given these findings, we sought to determine whether reducing the levels of Lck or Fyn altered the ability of a γδ T cell to become an IL-17- and/or IFNγ-producing effector cell. To accomplish this, we chose to use our γδTCR Tg mouse model as it generates, on the WT background, 30-fold more DN γδ T cells than non-γδTCR Tg mice [30]. This means that DN γδ T cells can be analyzed without the concern that purification by positive selection using an anti-TCRγδ mAb may crosslink the γδTCR and, in turn, pre-activate the γδ T cell. First, we determined whether there were any differences in the percentages of γδ T cells expressing CD122, CD27 or IL-23R among WT γδTCR Tg, Lck^+/−^ γδTCR Tg, and Fyn^+/−^ γδTCR Tg mice. We found that both Lck^+/−^ γδTCR Tg and Fyn^+/−^ γδTCR Tg mice had similar percentages of CD27^+^ and CD27^−^ γδ T cells as WT γδTCR Tg mice, but the percentages of CD27^+^ cells co-expressing CD122^+^ in both Lck^+/−^ γδTCR Tg and Fyn^+/−^ γδTCR Tg mice was reduced compared to WT γδTCR Tg mice (Fig. 5A). As CD122 expression by γδ T cells is induced when the γδTCR interacts with its ligand in the thymus [39], these results suggested that the selection and/or survival of CD122^+^ γδ T cells is impaired when Lck or Fyn levels are reduced. To evaluate IL-23R expression among WT, Lck^+/−^, and Fyn^+/−^ γδ T cells, we performed quantitative real-time RT-PCR analysis to detect transcription of *IL12RB1* and *IL23R*, which encode the two subunits of the IL-23R [44]. No significant differences were noted in the relative amounts of *IL12RB1* and *IL23R* transcripts among WT, Lck^+/−^, and Fyn^+/−^ γδ T cells (Fig. 5B), indicating that the selection and/or survival of γδ T cells with the potential to produce IL-17 is not affected when Lck or Fyn levels are reduced.

Next, we assessed cytokine production by WT, Lck^+/−^, and Fyn^+/−^ γδ T cells following CD3 crosslinking. Interestingly, we found that the percentages of WT, Lck^+/−^ and Fyn^+/−^ γδ T cells producing IFNγ at 16 h were equivalent to the percentages of CD122^+^ CD27^+^ γδ T cells in each mouse (Fig. 5A and C). It is also important to note that the level of IFNγ production, as measured by MFI, was 2 to 3-fold less in Lck^+/−^ and Fyn^+/−^ IFNγ^+^ γδ T cells than in WT IFNγ^+^ γδ T cells (Fig. 5C and data not shown). Moreover, when we compared the ability of WT, Lck^+/−^ and Fyn^+/−^ γδ T cells to differentiate into IL-17-producing cells at 16 h, we detected considerably fewer Fyn^+/−^ IL-17^+^ γδ T cells than Lck^+/−^ or WT IL-17^+^ γδ T cells (Fig. 5C). Taken together, these data indicated that reducing the levels of Fyn impacts the function of γδ T cells that have the potential to become either IL-17- or IFNγ-producing effector cells, while reducing the levels of Lck only impacts the function of γδ T cells that have the potential to become IFNγ-producing effector cells.

Because Lck^+/−^ and Fyn^+/−^ γδ T cells do not efficiently produce IFNγ, it was of interest to determine whether reducing Lck or Fyn levels also affected the ability of γδ T cells to produce other cytokines. To test this, we evaluated TNFα production by γδ T cells from Lck^+/−^ γδTCR Tg and Fyn^+/−^ γδTCR Tg mice, since γδ T cells, including those that produce IFNγ, have been shown to produce this cytokine [40]. As shown in Fig. 5D, we found that more γδ T cells from Lck^+/−^ γδTCR Tg and Fyn^+/−^ γδTCR Tg mice than from WT γδTCR Tg mice were producing TNFα. These data indicated that reducing Lck or Fyn levels does not impair the ability of γδ T cells to produce TNFα and suggested that the TCR signals required to activate the genetic program for IFNγ production are different than those for TNFα production.

## Discussion

Since SFKs have both positive and negative roles in receptor signaling, it has been postulated that these kinases function more like rheostats than on/off switches [45]. Our data support this idea, as changes in the cellular levels of Lck or Fyn at different stages in the life of a γδ lineage cell affected the strength of the γδTCR signaling response and, in turn, affected αβ/γδ lineage commitment, γδ T cell maturation and γδ T effector cell differentiation.

The expression levels of Lck and Fyn change during T cell development and maturation. Immature thymocytes (i.e., DN2 thymocytes), which have the potential to become either αβ or γδ lineage cells [26], [27], expressed relatively high levels of both Lck and Fyn. In thymic γδ lineage cells, these high levels of Lck and Fyn were maintained and, not until the γδ lineage cells were exported from the thymus, did their Lck and Fyn expression levels decrease. However, in immature αβ lineage cells, Lck and Fyn expression levels dramatically declined and, at the DP stage, their levels of Lck and Fyn were extremely low. The low SFK expression in DP thymocytes has also been reported by Olszowy et al. [46], who used quantitative Western blot analysis to measure Lck and Fyn protein levels in thymocyte subsets. Interestingly, Lck and Fyn levels were increased in TCR^hi^ DP (data not shown) and SP thymocytes compared to DP thymocytes, suggesting that positive selection upregulated both Lck and Fyn expression. It is important to note that mature SP thymocytes, after leaving the thymus, downregulated Lck and Fyn expression levels, but not to the levels observed for DP thymocytes nor to the extent observed between thymic and peripheral γδ T cells.

To investigate the importance of the quantitative difference in Lck and Fyn expression levels between thymic and peripheral γδ T cells, we used Lck^+/−^ and Fyn^+/−^ mice to study the effect of reducing Lck or Fyn expression levels on γδ T cell development and function. We chose to reduce, as opposed to eliminate, Lck and Fyn expression levels to prevent any compensatory action that one SFK may exhibit in the absence of the other. Although Fyn levels were reduced by 50% in αβ lineage cells from Fyn^+/−^ mice, they were only reduced by 40% in γδ lineage cells from the same mice. There are two possible explanations, which are not mutually exclusive, for why Fyn levels were not reduced to the expected 50% in γδ lineage cells from Fyn^+/−^ mice. First, the relatively high levels of Fyn in Fyn^+/−^ γδ thymocytes may be a result of selection, where only cells with high levels of Fyn survive and continue to mature. The second possibility is that the high Fyn levels in γδ lineage cells reflect high Fyn levels in a precursor population, such as DN2 thymocytes, which have the developmental potential to give rise to αβ and γδ lineage cells [26], [27]. Indeed, we found that Fyn expression was only reduced by ∼15% in DN2 thymocytes from Fyn^+/−^ mice. Therefore, even though we cannot rule out selection of γδ thymocytes with high levels of Fyn, it is conceivable that the relatively high levels of Fyn in γδ thymocytes from Fyn^+/−^ mice may be a direct result of the high levels of Fyn in Fyn^+/−^ DN2 thymocytes.

The high level of Fyn in thymic precursors highlights the importance of Fyn activity during an early stage of T cell development. As surface TCR complexes are not expressed at this stage, it is possible that Fyn is required for signaling through other receptors. One such receptor may be the IL-7 receptor (IL-7R), as Fyn has been shown to be recruited to this receptor [47], [48]. Given this association and that DN2 thymocytes require IL-7R expression and signaling for their survival and proliferation [49]–[53], it is possible that relatively high levels of Fyn are required for proper IL-7R signaling at this stage.

The first stage in T cell development where we observed regulation of γδTCR signal strength by Lck and Fyn is during αβ/γδ lineage commitment. We and others have previously demonstrated that TCR signal strength influences the αβ/γδ lineage decision, with a strong signal favoring γδ lineage commitment and a weak signal favoring αβ lineage commitment [29], [33], [54]. By fixing the specificity of the γδTCR, we were able to detect changes in the γδTCR signal response that were not apparent with a polyclonal γδTCR repertoire. Reducing the expression of Lck weakened γδTCR signal strength and resulted in a striking increase in the percentage and number of DP thymocytes in Lck^+/−^ γδTCR Tg mice compared to WT γδTCR Tg mice. Moreover, weakening of the γδTCR signal was confirmed by the finding that DN γδ thymocytes from Lck^+/−^ γδTCR Tg mice expressed higher levels of the γδTCR than WT DN γδ thymocytes. Together, these results indicated that relatively high levels of Lck are required to achieve the appropriate TCR signal response to support the γδ lineage choice. Conversely, reducing Fyn expression levels strengthened the γδTCR signaling response, as evidenced by the decrease, albeit not significant, in the number of DP thymocytes and the significant decrease in γδTCR surface expression on DN γδ thymocytes from Fyn^+/−^ γδTCR Tg mice. In contrast to the results of our previous study, in which γδ T cell fate was favored over αβ T cell fate when γδTCR signal strength was augmented [29], reducing Fyn expression had modest effects on the generation of αβ and γδ lineage cells. This difference may be attributed to the fact that Fyn expression was reduced by 35% in γδ thymocytes from Fyn^+/−^ γδTCR Tg mice (data not shown), suggesting that immature DN thymocytes expressing low levels of Fyn were unable to survive and develop into αβ or γδ lineage cells.

Our data also demonstrated that the alterations in γδTCR signal strength by reducing Lck or Fyn expression levels affected γδ T cell maturation in the thymus. When γδTCR signal strength was weakened by reducing Lck levels, there was a significant decrease in the number of DN γδ T cells in LNs despite normal numbers of γδ lineage cells present in the thymus. In support of a maturation defect, the frequency of recent thymic emigrants (CD24^+^ DN γδTCR^+^) was reduced in the LNs of Lck^+/−^ γδTCR Tg mice. Because of the reduction in thymic output, Lck^+/−^ γδ T cells seemingly underwent homeostatic proliferation in the periphery, evidenced by the increased frequency of γδ T cells with an activated/memory phenotype (CD44^+^ DN γδTCR^+^). On the other hand, strengthening the γδTCR signal response by reducing Fyn levels had little effect on thymic maturation. These data not only demonstrate that signaling by the γδTCR is required following γδ lineage commitment for γδ thymocyte maturation, but also indicate that the cellular levels of Lck in γδ thymocytes regulate the γδTCR signaling response at this developmental stage.

Unlike αβ thymocytes, γδ thymocytes do not need to encounter Ag to mature and emigrate to the periphery [39], [55]. However, a recent study demonstrates that Ag encounter in the thymus by γδ T cells controls their effector fate, with Ag-experienced γδ T cells expressing CD122 and preferentially producing IFNγ and Ag-naïve γδ T cells lacking CD122 expression and preferentially producing IL-17 [39]. In addition to CD122 expression, γδ T cell effector fate has been shown to segregate with CD27 expression, in that CD27^+^ γδ T cells produce IFNγ and CD27^−^ γδ T cells produce IL-17 [40]. We found that either weakening or augmenting the γδTCR signal response had an impact on the generation and/or survival of CD122^+^ CD27^+^ γδ T cells but not on the generation and/or survival of CD122^−^ CD27^+^ γδ T cells. Unfortunately, we do not know whether the defect lies in CD122^+^ γδ T cell selection, survival or both, as very few (<0.5%) CD122^+^ γδ T cells are detected in the thymus of WT γδTCR Tg, Lck^+/−^ γδTCR Tg, and Fyn^+/−^ γδTCR Tg mice (data not shown). Since Vγ6/Vδ1^+^ γδ T cells are normally generated in the fetal thymus [28], the paucity of CD122^+^ γδ thymocytes in WT γδTCR Tg mice may be due to decreased or negligible expression of the ligand for the Vγ6/Vδ1 γδTCR in the adult thymus. Nonetheless, we propose that weakening the γδTCR signal results in the generation of fewer CD122+ γδ thymocytes following Ag encounter in the thymus. However, due to homeostatic proliferation in the periphery, the size of the CD122^+^ γδ T cell pool is consequently increased. When the γδTCR signal is augmented, on the other hand, Ag encounter in the thymus may lead to negative selection and, in turn, fewer γδ thymocytes and peripheral γδ T cells expressing CD122. Surprisingly, in regards to γδ T cells with the potential to produce IL-17, we found that neither weakening nor augmenting γδTCR signal strength had an impact on their generation and/or survival, based on the comparable expression of *IL23R* and *IL12RB1* in γδ T cells from the three genotypes. These results indicate that γδ T cells destined to become IL-17 producers, unlike those destined to become IFNγ producers, are not dependent on Lck- or Fyn-mediated signaling for their generation and/or survival, and suggest that the choice to become an IFNγ effector versus an IL-17 effector is not a binary fate decision.

Consistent with their being multiple γδ effector fates is our finding that, following TCR stimulation, 10% of WT γδ T cells produce IFNγ, 5% produce IL-17 and 30% produce TNFα [a third of these also produce IFNγ, while none produce IL-17 (data not shown)]. Interestingly, the frequency of CD122^+^ γδ T cells but not CD27^+^ γδ T cells was found to be an indicator of IFNγ production by γδ T cells, regardless of whether the stimulated γδ T cells were from WT γδTCR Tg, Lck^+/−^ γδTCR Tg, and Fyn^+/−^ γδTCR Tg mice. While our finding is consistent with the findings of the recent study demonstrating that CD122 expression correlates with IFNγ production, it is not consistent with those of the other study demonstrating that CD27 expression correlates with IFNγ production. An explanation for the difference in results may be how, in each study, γδ T cells were stimulated. In our study, as well as that of Jensen et al. [38], γδ T cells were stimulated by crosslinking the TCR with anti-CD3 or anti-TCRδ mAbs. However, in the study of Ribot et al. [39], γδ T cells were first activated and expanded in the presence of anti-CD3 mAb and IL-2 for four days prior to re-stimulation with the phorbol ester, phorbol 12-myristate 13-acetate, and ionomycin. These data, taken together, suggest that CD122^+^ γδ T cells rapidly produce IFNγ following TCR activation, whereas CD27^+^ γδ T cells require multiple days post TCR activation to differentiate into IFNγ-producing cells. Notably, both weakening and augmenting γδTCR signal strength affected the proficiency by which γδ T cells produced IFNγ but not TNFα. This finding indicates that γδTCR signal strength regulates the quality of the cytokine effector response of stimulated γδ T cells. Moreover, having both weak and strong TCR signals leading to a similar outcome is not novel in T cell biology, as for example it well known that DP thymocytes that receive TCR signals that are too weak or too strong undergo apoptosis [reviewed in 56].

In summary, we have demonstrated that the cellular levels of Lck or Fyn regulate the strength of the γδTCR signaling response. Specifically, we found that reducing Lck levels, thereby weakening γδTCR signal strength, had profound effects on αβ/γδ lineage commitment, γδ thymocyte maturation, and the generation of IFNγ-producing effectors. In contrast, when Fyn levels were reduced, thus augmenting γδTCR signal strength, we observed defects in the generation of both IL-17- and IFNγ-producing effectors. Thus, this study has revealed that a relatively strong γδTCR signaling response is required following γδ lineage commitment for γδ T cell maturation and γδ T effector cell differentiation.

## Materials and Methods

### Ethics Statement

All research involving animals has been conducted according to the relevant national and international guidelines with respect to husbandry, experimentation and welfare. Mouse protocols were approved by the SUNY Upstate Medical University Committee on the Humane Use of Animals.

### Mice

C57BL/6 (wild-type or WT), B6.129S2-*Lck^tm1Mak^*/J (Lck^−/−^) and B6.129S7-*Fyn^tm1Sor^*/J (Fyn^−/−^) mice were originally purchased from The Jackson Laboratory (Bar Harbor, ME, USA). C57BL/6-Vγ6/Vδ1 γδTCR Tg mice (γδTCR Tg; line 134) [28] were kindly provided by P.E. Love (NICHD, Bethesda, MD, USA). Mice used in this study were bred and maintained in the Department of Laboratory Animal Resources at SUNY Upstate Medical University in accordance with the specifications of the Association for Assessment and Accreditation of Laboratory Animal Care. All mice were sacrificed at 6–8 weeks of age.

### Antibodies

Monoclonal antibodies (mAbs) used for flow cytometric analysis, γδ T cell separation and γδ T cell stimulation included anti-CD3 (145-2C11), anti-CD4 (RM4-5), anti-CD5 (53-7.3), anti-CD8α (53-6.7), anti-CD8β (53-5.8), anti-TCRγδ (UC7-13D5), anti-TCRβ (H57-597), anti-CD11b (M1/70), anti-CD16/CD32 (2.4G2), anti-CD19 (6D5), anti-IA^b^ (AF6-120.1), anti-CD24 (M1/69), anti-CD27 (LG.7F9), anti-CD44 (IM7), anti-CD122 (5H4), anti-NK1.1 (PK136), and anti-Ly6-G/Ly6-C (RB6-8C5), which were purchased from BD Pharmingen (San Jose, CA, USA), BioLegend (San Diego, CA, USA) or eBioscience (San Diego, CA, USA). mAbs used for intracellular (i.c.) flow cytometric analysis were Ki-67 (B56; BD Pharmingen), anti-Lck (3A5; Upstate Biotechnology, Billerica, MA, USA), anti-Fyn (FYN-59; BioLegend), anti-mouse IgG_2b_ (R12-3; BD Pharmingen), anti-IL-17A (TC11-18H10.1; BioLegend), anti-TNFα (MP6-XT22; BioLegend), and anti-IFNγ (XMG1.2; BD Pharmingen).

### *In Vitro* Stimulation of γδ T Cells

3×10^6^ lymph node (LN) cells from WT, Lck^+/−^ and Fyn^+/−^ γδTCR Tg mice were resuspended in RPMI 1640 supplemented with non-essential amino acids, L-glutamine, HEPES, sodium pyruvate, and penicillin/streptomycin (all from Invitrogen, Carlsbad, CA, USA) in addition to 10% FBS (Mediatech, Inc., Manassas, VA, USA), plated onto 1 or 5 µg/ml of immobilized anti-CD3 mAb or 5 µg/ml of immobilized hamster isotype control, and then cultured for 16 or 48 h at 37°C. 5 h prior to fixation, permeabilization, and i.c. staining with mAbs against IL-17A, TNFα and/or IFNγ, cells were treated with Brefeldin A and Monensin (eBioscience).

### Flow Cytometry

Flow cytometric analysis for surface antigens was performed by pre-incubating cells with the anti-CD16/CD32 mAb for at least 10 min to block non-specific binding of immunoglobulins to Fc receptors followed by staining with fluorochrome-conjugated Abs against the various surface antigens. I.c. staining for Ki-67 was performed according to the manufacturer's instructions (BD Pharmingen). I.c. staining for Lck and Fyn in addition to IL-17A, TNFα and IFNγ was performed by first fixing cells in a final concentration of 1.5% formaldehyde for 10 min at 37°C. Fixed cells were then stained for surface antigens, permeabilized with Perm/Wash Buffer (BD Pharmingen) for 20 min at 4°C, and then stained with mAbs against the appropriate proteins. The mAbs specific for IL-17A, TNFα and IFNγ were directly conjugated to a fluorochrome, while FITC-conjugated anti-mouse IgG_2b_ Ab was used as a secondary reagent to detect the anti-Lck mAb or the anti-Fyn mAb. For all experiments, 0.1–2×10^6^ cells were collected on a LSR II using FACSDiva software (BD Immunocytometry Systems, San Jose, CA, USA) and analyzed using FlowJo software (Tree Star Inc., Ashland, OR, USA). Dead cells were excluded from analysis based on forward and side scatter profiles.

### Cell Separation

DN γδ T cells were purified by negative selection from the LNs of WT γδTCR Tg, Lck^+/−^ γδTCR Tg and Fyn^+/−^ γδTCR Tg mice using the MACS magnetic bead separation system (Miltenyi Biotec, Auburn, CA, USA). LN cells were stained for 10 min at 4°C with a panel of FITC-labeled mAbs containing anti-CD19, anti-TCRβ, anti-CD4, anti-CD8, anti-IA^b^ and anti-DX5 mAbs. Cells were washed, incubated with anti-FITC MACS beads for 15 min at 4°C, and then separated on an autoMACS cell separator, according to manufacturer's directions. The purity of the resulting DN γδ T cell populations were typically ≥99%.

### RT-PCR Analysis

RNA was extracted from purified DN γδ T cells using the Qiagen RNeasy kit (Valencia, CA, USA). cDNA was then synthesized using Invitrogen's SuperScript® First-Strand Synthesis System. Quantitative real-time RT-PCR analysis was performed using a Bio-Rad iQ™5 Real-time PCR machine (Hercules, CA, USA) according to manufacturer's directions. All of the primer sets for the quantitative real-time RT-PCR analysis, which included *GAPDH*, *IL12RB1*, and *IL23R*, along with the SYBR Green PCR Master Mix were purchased from SABiosciences (Frederick, MD, USA).

### Statistical Analysis

Data are presented as mean ± SEM. Student's t-test was used for all statistical comparisons (Graph Pad Prism or Microsoft Excel software) except for the one evaluating Fyn levels in αβ lineage cells, γδ lineage cells and DN2 thymocytes from Fyn^+/−^ mice, in which a χ^2^ test (Microsoft Excel software) was used. *p* values less than or equal to 0.05 were considered statistically significant.
